# NAD+ Modulates the Proliferation and Differentiation of Adult Neural Stem/Progenitor Cells via Akt Signaling Pathway

**DOI:** 10.3390/cells11081283

**Published:** 2022-04-09

**Authors:** Xiaoli Huang, Hongfeng Guo, Xuejun Cheng, Jinyu Zhang, Wenzheng Qu, Qianyun Ding, Qihang Sun, Qiang Shu, Xuekun Li

**Affiliations:** 1 The Children’s Hospital, National Clinical Research Center for Child Health, Zhejiang University School of Medicine, Hangzhou 310052, China; xiaolihuang@zju.edu.cn (X.H.); hongfeng_guo@zju.edu.cn (H.G.); xuejun_cheng@zju.edu.cn (X.C.); jinyu_zhang@zju.edu.cn (J.Z.); quwz@zju.edu.cn (W.Q.); dingqianyun11@zju.edu.cn (Q.D.); sunqihang@zju.edu.cn (Q.S.); 2 The Institute of Translational Medicine, School of Medicine, Zhejiang University, Hangzhou 310029, China; 3 Zhejiang University Cancer Center, Zhejiang University, Hangzhou 310029, China

**Keywords:** adult neural stem/progenitor cells, proliferation, differentiation, NAD+, Akt pathway

## Abstract

Nicotinamide adenine dinucleotide hydrate (NAD+) acts as the essential component of the tricarboxylic citric acid (TCA) cycle and has important functions in diverse biological processes. However, the roles of NAD+ in regulating adult neural stem/progenitor cells (aNSPCs) remain largely unknown. Here, we show that NAD+ exposure leads to the reduced proliferation and neuronal differentiation of aNSPCs and induces the apoptosis of aNSPCs. In addition, NAD+ exposure inhibits the morphological development of neurons. Mechanistically, RNA sequencing revealed that the transcriptome of aNSPCs is altered by NAD+ exposure. NAD+ exposure significantly decreases the expression of multiple genes related to ATP metabolism and the PI3k-Akt signaling pathway. Collectively, our findings provide some insights into the roles and mechanisms in which NAD+ regulates aNSPCs and neuronal development.

## 1. Introduction

Adult neural stem/progenitor cells (aNSPCs) can self-renew and display a multipotent capability to generate astrocytes and neurons upon differentiation. aNSPCs generated in newborn neurons can become mature neuronal cells and are involved in physiological functions, including hippocampus-dependent learning and memory [1]. Diverse mechanisms including an enriched environment, niche, genetics and epigenetics are involved in regulating the proliferation and differentiation of aNSPCs [2,3,4,5,6,7,8,9,10,11,12,13,14]. The dysregulation of adult neurogenesis also involves neurological disorders such as stroke, Rett syndrome, Alzheimer’s disease and depression [15,16,17].

As a key redox cofactor for metabolism and ATP production, nicotinamide adenine dinucleotide hydrate (NAD+) plays essential roles in the tricarboxylic citric acid (TCA) cycle and is involved in bioenergetics, mitochondrial homeostasis, inflammation, cell survival and senescence [18]. The treatment with the NAD+ precursor nicotinamide riboside (NR) prevents the senescence of stem cells and improves life span [19,20]. During normal brain aging, the level of NAD+ showed a remarkable decrease, which also was observed in neurodegenerative diseases [18,21,22,23]. Consistently, the supplementation of NR increased the level of NAD+, promoted the proliferation of neural progenitor cells and neurogenesis and improved the cognitive function of neurodegenerative animal models [24]. In addition, NAD+ exposure inhibited cancer cell growth and regulated gene expression via modulating DNA methylation [25]. 

In the present study, we studied the roles of nicotinamide adenine dinucleotide hydrate (NAD+) in regulating aNSPCs. We observed that NAD+ exposure inhibited the proliferation and neuronal differentiation but also promoted the glial differentiation of aNSPCs. NAD+ exposure also induced the apoptosis of aNSPCs and inhibited the morphological development of neurons. Mechanistically, NAD+ exposure reduced the expression of multiple genes relating to the Akt signaling pathway. Our findings suggest that NAD+ could have multi-faceted functions in the neuronal system.

## 2. Materials and Methods

### 2.1. The Isolation and Culturing of Adult Neural Stem/Progenitor Cells and Hippocampal Neurons

The isolation of adult neural stem/progenitor cells (aNSPCs) was performed as described previously [3,7]. Briefly, the forebrains of adult mice (C57BL6, age of postnatal 8–10 weeks) were cut into small pieces followed by enzymatic digestion, filtering and centrifugation. aNSPCs were cultured with DMEM/F-12 medium containing 2% B27 (Cat# 12587-010, Thermo Fisher Scientific, Grand Island, NY, USA), 20 ng/mL EGF (Cat# 100-15, Peprotech, Rocky Hill, NJ, USA), 20 ng/mL FGF-2 (Cat# 100-18B, Peprotech), 2 mM glutamine (Cat# 5030-149, Thermo Fisher Scientific) and 1% antibiotic–antimycotic (Cat# 15140-122, Thermo Fisher Scientific) at 37 °C in a humidified incubator containing 5% CO_2_. The medium was half replaced every other day. 

The isolation and culture of primary neurons were performed as previously described [4]. In brief, the hippocampi of embryonic day 16–18 (E16–18) mouse pups were dissected, cut in to small pieces followed by digestion with 0.25% Trypsin for 12 min, which were further treated with DNase 1 for 30 s–1 min. Cells were seeded on cell climbing slices or Poly-D-Lysine (5 μg/mL, Cat# P0899-10, Sigma, Saint Louis, MO, USA) coated plates. Approximately 1 × 10^5^ cells per well were seeded for a slice, and approximately 1.5 million cells were seeded per well for a 6-well-plate. After growing in the plating medium for 4 h, which comprised MEM (Cat# 11095-080, Gibco, Carlsbad, CA, USA), 10% FBS (10091-148, Gibco), 1%L-Glu (5030-149, Gibco), 1% sodium pyruvate (Cat# 11360-070, Gibco), 0.45% D-Glucose (Cat# 0188, Amresco, Radnor, PA, USA), the medium was replaced with a medium that consisted of neurobasal (Cat# 21103-049, Gibco), 0.25% L-Glu (Cat# 25030-149, Gibco), 0.125% GlutaMax (Cat# 35050061, Thermo Fisher Scientific) and 2% B27 (Cat# 17504-044, Gibco). Half of the medium was replaced every three days.

### 2.2. NAD+ Exposure and the Proliferation and Differentiation Assays of aNSPCs

β-nicotinamide adenine dinucleotide hydrate (NAD+) was dissolved with grade water and was applied to cells at the final concentration of 1 mM or 2 mM. To analyze the proliferating ability of aNSPCs, NAD+ was supplemented with aNSPCs for 40 h. We added 5 µM BrdU and aNSPCs were further cultured for 8 h. To analyze the differentiation capability of aNSPCs, NAD+ together with 1 µM retinoic acid (Cat# R-2625, Sigma) and 5 µM forskolin (Cat# F-6886, Sigma) was supplemented, and aNSPCs were further cultured for 48 h. At the scheduled time points, the cells were fixed with 4% paraformaldehyde for 30 min and immunostaining assays were performed. 

### 2.3. Neurospheres Formation Assay

The cultured aNSPCs were pipetted and filtered out with a 70 μm cell strainer (Cat# 352350, FALCON, Durham, NC, USA) to obtain the single-cell suspension at approximately one cell in a 100 μL medium. For the NAD+ exposure group, NAD+ was added at the final concentration of 1 mM. Then, 100 μL suspension was sequentially added per 96-well plate. Each well plate was observed the next day, and only the wells with single cells were marked. The images were captured on days 7 and 14 using a Nikon inverted microscope. For cells to be analyzed on day 14, 100 μL fresh medium was added to each well of the 96-well plate at day 7. The cross-sectional area of the neurospheres was analyzed using ImageJ software.

### 2.4. Transfection of Neuronal Cells and Sholl Analysis

Half of the volume of medium was replaced with fresh medium 6 h before the transfection. Five hours later, the maintaining medium was replaced with neurobasal medium, and the maintaining medium was stored at 4 °C. Additionally, 3 μg plasmid vector was mixed with 4 μL lipo2000 (Thermo Fisher Scientific) in 200 μL neurobasal medium. Thirty minutes later, the mixture was added to the cultured cells, and the medium was completely replaced with fresh maintaining medium 4 h later. 

For Sholl analysis, images of individual neurons were converted to 8-bit images, and NeuronJ was used to manually track dendritic arborization and automatically analyze the length and branch number of individual cells. Traced images were then imported into Fiji and were converted into 8-bit binary images. The soma center and neurite termination points were marked using the straight tool. Sholl analysis was carried out using the Fiji plugins Sholl Analysis (available online: http://fiji.sc/Sholl_Analysis, version 4.0.1) [26] at 20 μm intervals to a maximum radius of 1000 μm. The intersections at each concentric circle were counted and were plotted using the number of intersections over the distance from soma center.

### 2.5. ATP, NADP+ and NADPH Level Measurement

The level of ATP was analyzed following the manufacturer’s protocol (Cat#S0027, Beyotime Biotechnology, Shanghai, China). In brief, cultured cells were harvested after treated with 1 mM NAD+ for 48 h and were completely resuspended with ATP lysis buffer. After centrifuged at 14,000 rpm, 4 °C for 5 min, the supernatant and the standard samples were added to an opaque 96-well plate and were mixed with the substrate solution at room temperature. The luminescence was recorded using a SpectraMax M5 illuminometer (Molecular Devices, San Jose, CA, USA). The concentration was calculated according to the standard curve and then divided by the protein concentration.

The measurement of NADP+ and NADPH levels was performed following the manufacturer’s protocols (Cat# S0179, Beyotime Biotechnology). In brief, cultured cells were harvested after being treated with 1 mM NAD for 48 and lysed with extraction buffer completely. After being centrifuged at 14,000 rpm, 4 °C for 5 min, the supernatant and the standard samples were added to an opaque 96-well plate and mixed with the G6PDH working solution for 10 min at room temperature. Moreover, 10 μL chromogen reagent was added for formazan formation. The absorbance at 450 nm was recorded using a SpectraMax M5 illuminometer (Molecular Devices). The concentration was calculated according to the standard curve and then divided by the protein concentration.

### 2.6. Western Blot Assay

Cell samples were washed with PBS and homogenized in RIPA lysis buffer (Cat# ab156034, Abcam, Cambridge, MA, USA) containing 1X protease inhibitor cocktail (Cat# 04693124001, Sigma). Moreover, 20 μg of each sample was subjected to SDS-PAGE, transferred to nitrocellulose membranes followed by the blocking with PBS containing 5% skimmed milk for 1 h, and incubated with primary antibodies overnight at 4 °C. The following primary antibodies were used: anti-mouse Tuj1 (Cat# G712A, Promega, Wisconsin, WI, USA), anti-mouse GFAP (Cat# 3670, Cell Signaling technology, Danvers, MA, USA), anti-mouse Akt (Cat# 4691, Cell Signaling Technology), anti-mouse phospho-Akt (Ser473) (Cat# 4060, Cell Signaling Technology), anti-mouse Nestin (Cat# 556309, BD Biosciences, Lake Franklin, NJ, USA), anti-rabbit Sox2 (Cat# ab97959, Abcam) and anti-mouse GAPDH (Cat# AM4300, Thermo Fisher Scientific). The second day, HRP-conjugated secondary anti-mouse and anti-rabbit antibodies were incubated for 1 h at room temperature. The images were visualized and captured using a Molecular Imager Imaging System (Tanon, Shanghai, China). The relative level of signal intensity was normalized to GAPDH and was analyzed with the ImageJ software.

### 2.7. Immunostaining and Cell Quantification

The immunofluorescence staining and cell quantification were carried out as described previously [2,27]. Briefly, the cell samples were washed with PBS for 30 min followed by blocking with PBS (3% normal goat serum, 0.1% triton X-100 in PBS) for 1 h at room temperature. Primary antibodies were applied overnight at 4 °C. The following primary antibodies were used: anti-rat BrdU (Cat# ab6326, Abcam), anti-mouse GFAP (Cat# Z0334, DAKO, Santa Clara, CA, USA), anti-mouse Tuj1 (Cat# G712A, Promega), anti-rabbit Caspase 3 (Cat# AB3623, Millipore, Boston, MA, USA) and anti-mouse MAP2 (Cat# M9942, Sigma). The second day, fluorophore-conjugated secondary antibodies were applied for 1 h at room temperature. Images were captured and the numbers of BrdU^+^, Tuj1^+^, GFAP^+^ and Caspase3^+^ cells were quantified using ImageJ software (NIH). 

### 2.8. Total RNA Isolation, Reverse Transcription and Quantitative Real-Time PCR

Total RNA was extracted with TRIzol reagent (Cat# 15596018, Thermo Fisher Scientific) following the manufacturer’s protocol. For reverse transcription, 500 ng total RNA was used. PCR reactions were carried out in triplicate using power SYBR Green PCR master Mix (Cat# Q71502,Vazyme, Nanjing, China) and the ^∆∆^Ct method was used to analyze the results. The details of the used primers can be found in Supplemental Table S2.

### 2.9. RNA-Seq and Data Analysis

RNA-seq and data analysis were performed as described previously [3]. In brief, all samples were assessed with NanoDrop 2000 (Thermo Fisher Scientific), and the RNA integrity value (RIN) was determined with the RNA Nano 6000 Assay Kit of the Bioanalyzer 2100 system (Agilent Technologies Inc.,Palo Alto, CA, USA). A total amount of 3 µg of RNA per sample was used as input. Sequencing libraries were generated, and index codes were added to attribute sequences to each sample using NEBNext^®^ UltraTM RNA Library Prep Kit for Illumina^®^ (NEB, Ipswich, MA, USA). The generated raw reads of fastq format were processed, and clean reads were mapped to mm10 using Hisat2 v2.0.5. Only uniquely mapped reads were used for further analysis, and FeatureCounts v2.0.1 was used to count the read numbers mapped to each gene. 

### 2.10. Statistical Analysis

Data are presented as the mean ± SEM. Statistical analysis between group differences was performed with the two-tailed unpaired Student’s t test using Graph Prism software (version 9.0; GraphPad, San Diego, CA, USA). *p* < 0.05 was considered statistically significant. Replicate information is indicated in the figure legends.

## 3. Results

### 3.1. NAD+ Regulates the Proliferation and Differentiation of Adult Neural Stem/Progenitor Cells

**Figure 1:**
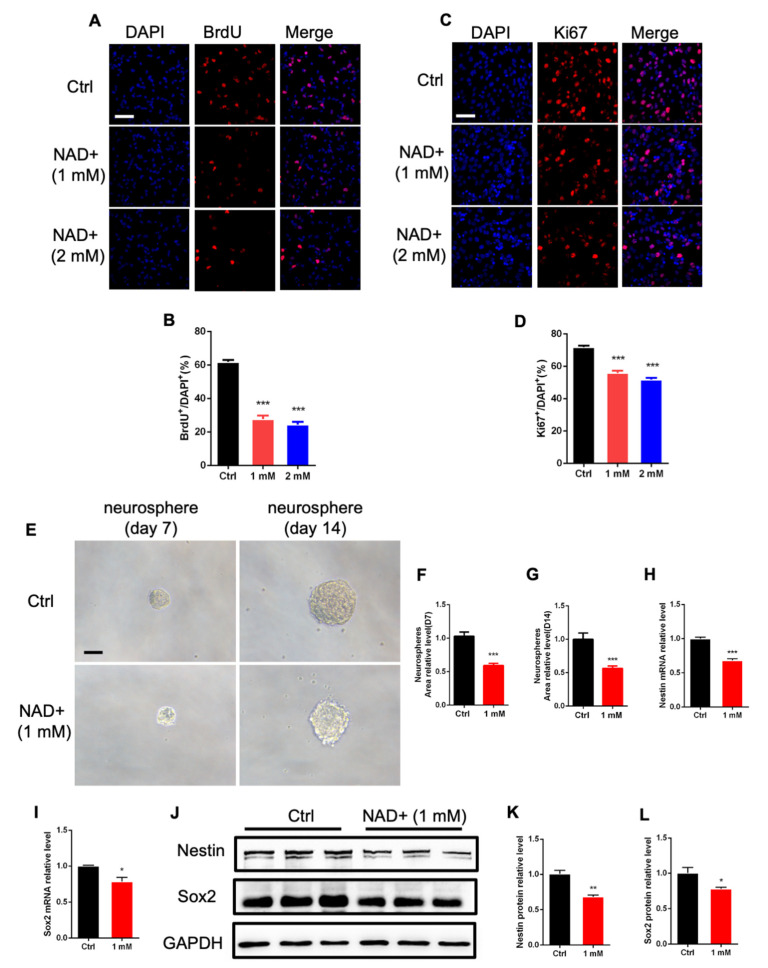
NAD+ exposure affects the proliferation of adult neural stem/progenitor cells in vitro. (**A**) Representative images of BrdU immunostaining with control (Ctrl) and NAD+-treated adult neural stem/progenitor cells (aNSPCs). Scale bar, 50 μm. (**B**) Quantification results show that NAD+ exposure decreased the percentages of BrdU^+^ cells under proliferation conditions. Data are presented as the mean ± SEM. *n* = 3 independent experiments, unpaired *t*-test. (**C**) Representative images of Ki67 immunostaining with Ctrl and NAD+-treated aNSPCs. Scale bar, 50 μm. (**D**) Quantification results show that NAD+ exposure decreased the percentages of Ki67^+^ cells. Data are presented as the mean ± SEM. *n* = 3 independent experiments, unpaired *t*-test. (**E**) Representative images of neurospheres derived from single aNSPCs. aNSPCs were supplemented with 1 mM NAD+ and analyzed at days 7 and 14. Scale bar: 50 μm. (**F**,**G**) Quantification results show that NAD+ exposure decreased the size of neurospheres at day 7 (**F**) and day 14 (**G**). Data are presented as the mean ± SEM. *n* = 30 neurospheres for Ctrl and NAD+ exposure groups at each timepoint. unpaired *t*-test. (**H**,**I**) The relative mRNA levels of *Nestin* and *Sox2* of Ctrl and NAD+ supplemented aNSPCs. Data are presented as the mean ± SEM. *n* = 3 independent experiments, unpaired *t*-test. (**J**–**L**) Western blot assay (**J**) and quantification results (**K**,**L**) show that NAD+ exposure decreased the level of *Nestin* and *Sox2*. GAPDH was used as an internal control. Data are presented as the mean ± SEM. *n* = 3 independent experiments, unpaired *t*-test; *, *p* < 0.05; **, *p* < 0.01; ***, *p* < 0.001.

To examine the roles of NAD+ in regulating the proliferation of neural stem/progenitor cells (aNSPCs), we first isolated aNSPCs from the forebrain of adult mice and cultured in vitro. Immunofluorescence staining results show that aNSPCs were positive for neural stem/progenitor cells markers Nestin and Sox2 (Supplemental Figure S1A) and that upon differentiation, aNSPCs generated neurons and astrocytes (Supplemental Figure S1B). aNSPCs were supplemented with NAD+ through a series of doses, and the BrdU incorporation assay was performed. We observed that 1 mM and 2 mM NAD+ exhibited the most significant effects based on BrdU immunostaining (Supplemental Figure S1C). Therefore, we adopted the doses of 1 mM and 2 mM in the following assays. Immunofluorescence staining and quantification results show that NAD+ exposure significantly reduced the percentages of BrdU^+^ cells (Figure 1A,B) and Ki67^+^ cells compared to the control group (Figure 1C,D). Furthermore, we performed the neurosphere assay and observed that 1 mM NAD+ exposure significantly reduced the size of neurospheres at day 7 and day 14 (Figure 1E–G). In addition, NAD+ exposure also led to a significant decrease in neural stem/progenitor cell markers Nestin and Sox2 at mRNA and protein levels, respectively (Figure 1H–L, Supplemental Figure S2A). These results suggest the capabilities of self-renewal and the multipotency of cultured aNSPCs. 

**Figure 2:**
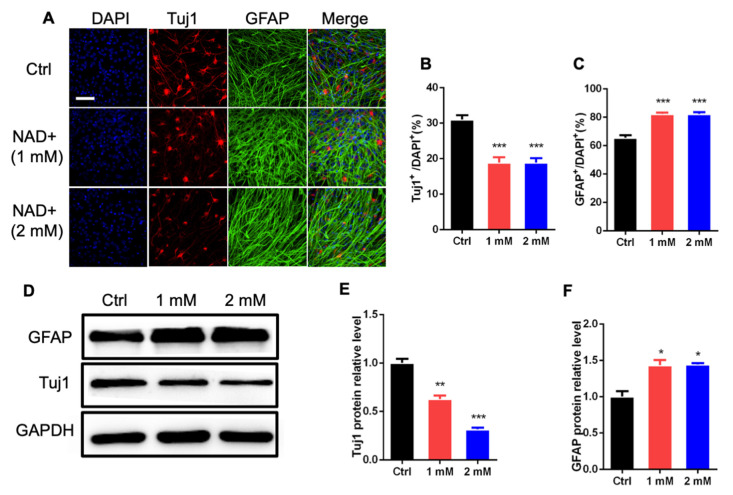
NAD+ exposure regulates the differentiation of aNSPCs in vitro. (**A**) Representative immunostaining images of neuronal marker β-III tubulin (Tuj1) and astrocytic marker glial fibrillary acidic protein (GFAP) with Ctrl and NAD+-treated aNSPCs under differentiation conditions. Scale bar, 50 μm. (**B**,**C**) Quantification results show that NAD+ exposure decreased the percentage of Tuj1^+^ cells but increased the percentages of GFAP^+^ cells under differentiation conditions. Data are presented as the mean ± SEM. *n* = 3 independent experiments, unpaired *t*-test. (**D**–**F**) Western blot assay (**D**) and quantification results (**E**,**F**) show that NAD+ exposure decreased the level of Tuj1, but increased the level of GFAP. GAPDH was used as an internal control. Data are presented as the mean ± SEM. *n* = 3 independent experiments, unpaired *t*-test; *, *p* < 0.05; **, *p* < 0.01; ***, *p* < 0.001.

We then analyzed the effects of NAD+ on the differentiation of aNSPCs. Immunofluorescence staining and quantification results show that NAD+ treatment significantly reduced the percentage of newborn neurons (Tuj1^+^), but increased the percentage of astrocytes (GFAP^+^) upon the differentiation of aNSPCs (Figure 2A–C). Western blot assay results also show that NAD+ exposure remarkably decreased the level of Tuj1 but increased the level of GFAP compared to the control group (Figure 2D–F). RT-PCR assay results also show that NAD+ exposure significantly increased the mRNA levels of astrocyte markers S100β and Glt1 (Supplemental Figure S2B,C). Collectively, these results suggest that NAD+ regulates the proliferation and differentiation of aNSPCs. 

### 3.2. NAD Regulates the Morphological Development of the aNSPCs-Derived Neuron and Hippocampal Neuron

**Figure 3:**
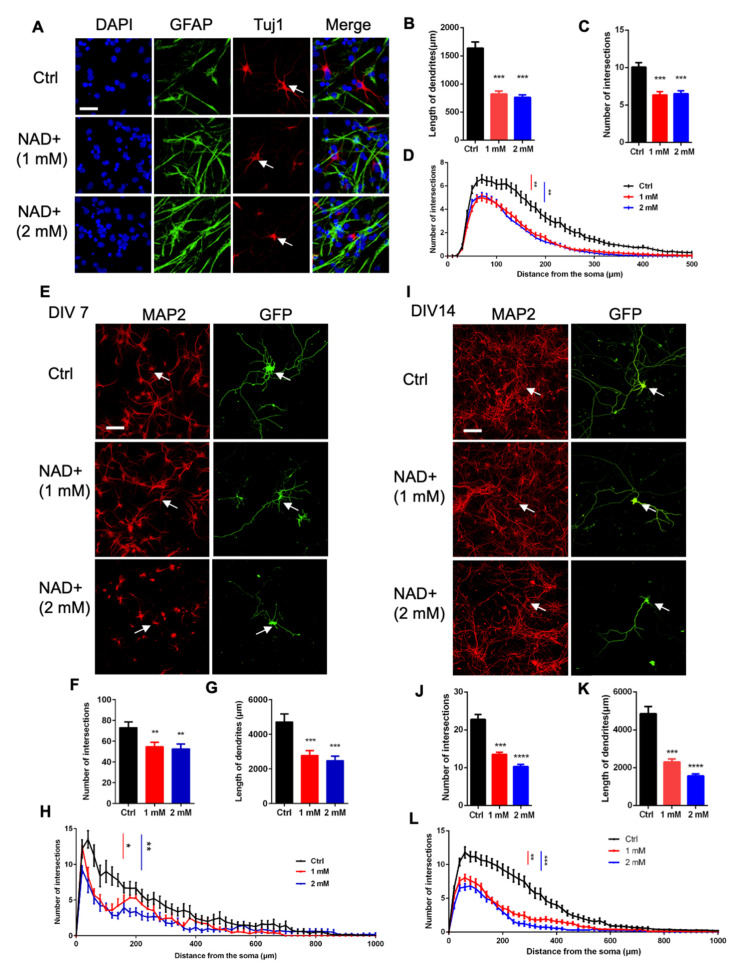
NAD+ exposure inhibits the morphological development of neurons. (**A**) Representative images of Tuj1 immunostaining with Ctrl and NAD+-treated aNSPCs. Scale bar, 50 μm. (**B**,**C**) The quantification results show that the NAD+ exposure significantly decreased the dendritic length (**B**) and the intersection number (**C**) of newborn neurons derived from aNSPCs. Data are presented as the mean ± SEM. Unpaired *t*-test. *n* = 30 neurons were analyzed in each group. (**D**) Sholl analysis results show that NAD+ exposure reduced the dendritic complexity of aNSPCs-derived neurons compared to Ctrl group. Data are presented as the mean ± SEM. Unpaired *t*-test. *n* = 30 neurons were analyzed in each group. (**E**) Representative images of MAP2 immunostaining with hippocampal neurons treated with NAD+. The cultured neurons were transfected with the GFP plasmid at 4 days in vitro (DIV 4). At DIV 5, NAD+ was supplemented at the final concentration of 1 mM and 2 mM. Forty-eight hours later, MAP2 immunostaining and morphological assay on the cells were performed. Scale bar, 50 μm. (**F**,**G**) The quantification results show that NAD+ exposure reduced the dendritic complexity of hippocampal neurons compared to the control group. Data are presented as the mean ± SEM. Unpaired *t*-test. *n* = 30 neurons were analyzed in each group. (**H**) Sholl analysis results showed that the NAD+ exposure reduced the dendritic complexity of hippocampal neurons compared to control group. Data are presented as the mean ± SEM. Unpaired *t*-test. *n* = 30 neurons were analyzed in each group. (**I**) Representative images of MAP2 immunostaining with hippocampal neurons treated with NAD+. The cultured neurons were transfected with GFP plasmid at 14 days in vitro (DIV 14). At DIV 15, NAD+ was supplemented at the final concentration of 1 mM and 2 mM. Cells were performed MAP2 immunostaining and morphological assay at DIV 17. Scale bar, 50 μm. (**J**,**K**) The quantification results show that NAD+ exposure reduced the intersection numbers (**J**) and dendritic length (**K**) of hippocampal neurons compared to control group. Data are presented as the mean ± SEM. Unpaired *t*-test. *n* = 30 neurons for Ctrl and 1 mM groups and *n* = 25 neurons for 2 mM group were analyzed. (**L**) Sholl analysis results show that NAD+ exposure reduced the dendritic complexity of hippocampal neurons compared to control group. Data are presented as the mean ± SEM. Unpaired *t*-test; *, *p* < 0.05; **, *p* < 0.01; ***, *p* < 0.001; ****, *p* < 0.0001.

We first analyzed the effects of NAD+ on neuronal development. Representative immunostaining images showed that NAD+ exposure affected the morphological development of newborn neurons generated upon the differentiation of aNSPCs (Figure 3A). Sholl analysis showed that NAD+ exposure significantly decreased the dendritic length and numbers of intersections of newborn neurons (Figure 3B–D).

We then isolated neurons from the hippocampal tissues of embryonic mice and immunostaining with the neuronal marker MAP2 antibody indicated the homogeneity of neuronal cells (Figure 3E). To trace the dendrites of neurons, we performed a transfection of GFP expressing plasmid (at DIV 4 and DIV 14). Twenty-four hours later (DIV 5 and DIV 15), hippocampal neurons were exposed to 1 mM and 2 mM NAD+ for 48 h, and cells were harvested at DIV 7 and DIV 17, respectively. We observed that NAD+ exposure led to a significant decrease in the intersection number and the dendritic length of hippocampal neurons at DIV 7 (Figure 3E–H) and DIV 17 (Figure 3I–L). Collectively, these results indicate that NAD+ exposure inhibits the morphological development of neurons.

### 3.3. NAD+ Exposure Induces Apoptosis of aNSPCs

**Figure 4:**
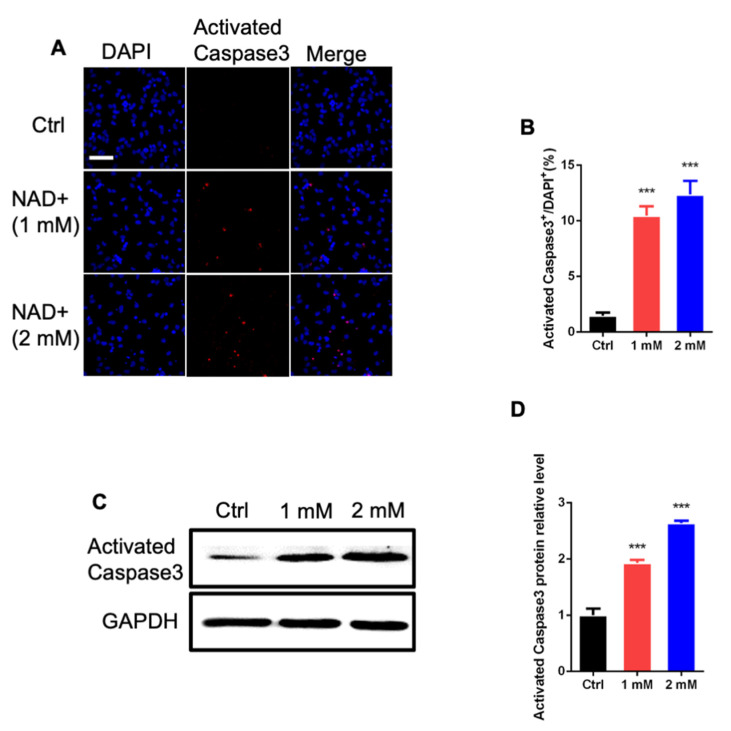
NAD+ exposure induces the apoptosis of aNSPCs. (**A**) Representative images of activated caspase3 immunostaining with Ctrl and NAD+-treated aNSPCs under proliferation conditions. Scale bar, 50 μm. (**B**) Quantification results show that NAD+ exposure increased the percentage of activated caspase3^+^ cells. Data are presented as the mean ± SEM. *n* = 3 independent experiments, unpaired *t*-test. (**C**,**D**) Western blot assay (**C**) and quantification results (**D**) show that NAD+ exposure increased the protein level of activated caspase3. GAPDH was used as an internal control. Data are presented as the mean ± SEM. *n* = 3 independent experiments, unpaired *t*-test; ***, *p* < 0.001.

We then examined whether NAD+ exposure affected the survival of aNSPCs. Immunofluorescence staining and quantification results show that NAD+ exposure significantly increased the percentage of activated Caspase3^+^ cells compared to the control group (Figure 4A,B). The Western blot assay results also show that NAD+ exposure remarkably increased the level of activated Caspase3 compared to the control group (Figure 4C,D). Together, these results suggest that NAD+ regulates the survival of newborn neurons. 

### 3.4. NAD+ Exposure Alters the Transcriptome of aNSPCs under Proliferating and Differentiation Conditions

**Figure 5:**
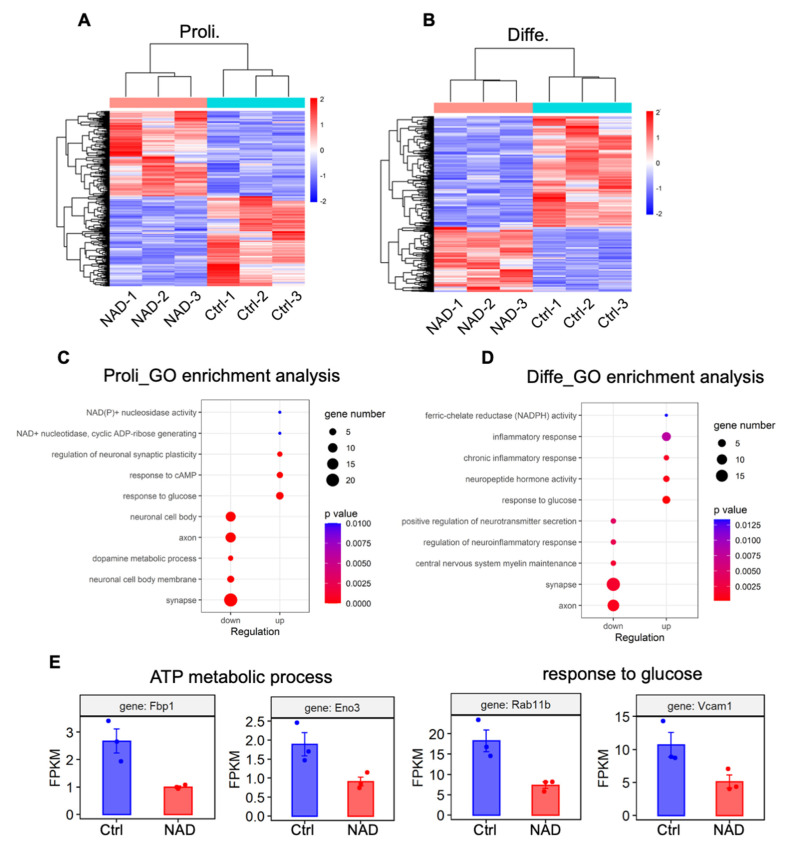
NAD+ exposure alters the transcriptome of aNSPCs under proliferating and differentiation conditions. (**A**,**B**) Heatmap showing the differentially expressed genes (DEGs) in Ctrl and NAD+ exposure groups under proliferating (**A**) and differentiation (**B**) conditions. Three biological repeating samples of Ctrl and NAD+ exposure (1 mM) were adopted for RNA sequencing. The significance of expression was determined by the |FC| > 2 and *p*-value < 0.05. (**C**) GO enrichment analysis of the up-regulated and down-regulated genes induced by NAD+ exposure (proliferating condition). The significance of expression was determined by |FC| > 2 and *p*-value < 0.05. (**D**) GO enrichment analysis of the up-regulated and down-regulated genes induced by NAD+ exposure (differentiation condition). The significance of expression was determined by |FC| > 2 and *p*-value < 0.05. (**E**) FPKM values of several genes related to ATP metabolic process and response to glucose.

To determine the mechanisms by which NAD+ regulates aNSPCs, we performed RNA-seq with proliferating and differentiated aNSPCs. The RNA-seq data analysis revealed 369 and 504 differentially expressed genes (DEGs) under proliferating and differentiation conditions, respectively (Figure 5A,B, Supplemental Table S1). Under proliferating conditions, among 369 DEGs, 180 genes were up-regulated and 189 genes were down-regulated. Under differentiation conditions, among 504 DEGs, 186 genes were up-regulated and 318 genes were down-regulated (Supplemental Table S1). Gene ontology (GO) analysis showed that up-regulated genes were enriched for terms including response to glucose and NAD(P)+/NADPH activity, whereas down-regulated genes were enriched for terms including synapse and axon development under proliferating and differentiation conditions (Figure 5C,D). In terms of consistency, the FPKM values of a few genes relating to ATP metabolic process (Fbp1 and Eno3) and the response to glucose (Rab11b and Vcam1) significantly decreased in the NAD+ group compared to that of the control group (Figure 5E). 

### 3.5. NAD+ Exposure Alters Akt Signaling Pathway

**Figure 6:**
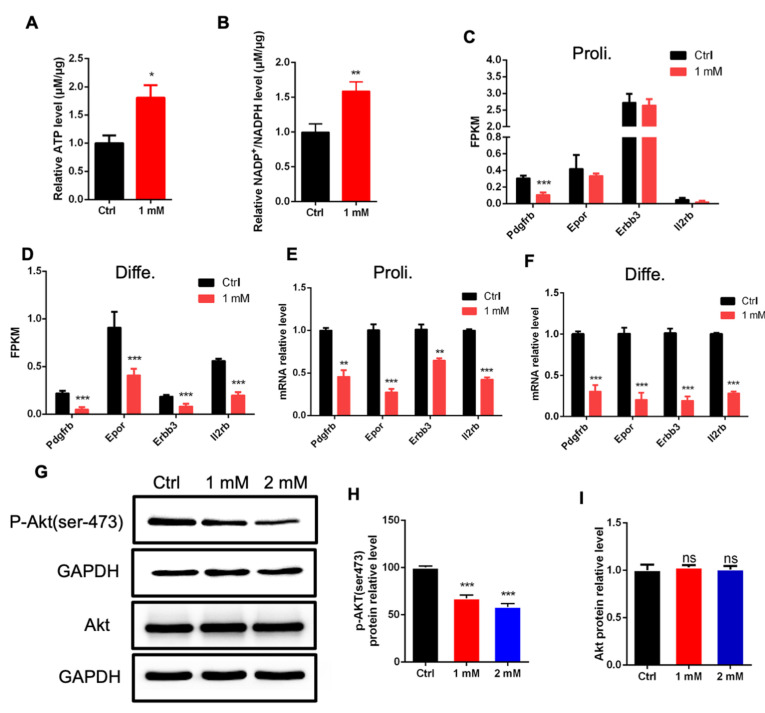
NAD+ exposure alters the PI3k-Akt signaling pathway. (**A**) ELISA results showing the increased level of ATP induced by NAD+ exposure compared to Ctrl group. Proliferating aNSPCs were cultured with 1 mM NAD+ supplemented for 48 h. Data are presented as the mean ± SEM. *n* = 3 independent experiments, unpaired *t*-test. (**B**) NAD+ exposure increased the levels of NADP^+^ and NADPH. Proliferating aNSPCs were cultured with 1 mM NAD+ supplemented for 48 h. Data are presented as the mean ± SEM. *n* = 3 independent experiments, unpaired *t*-test. (**C**) FPKM values of multiple genes relating to PI3k-Akt signaling pathway (proliferating condition). (**D**) FPKM values of multiple genes relating to PI3k-Akt signaling pathway (differentiation condition). (**E**) The relative mRNA levels of *Pdgfrb*, *Epor*, *Erbb3* and *Il2rb* in aNSPCs treated with NAD+ (proliferating condition). Data are presented as the mean ± SEM. *n* = 3 independent experiments, unpaired *t*-test. (**F**) The relative mRNA levels of *Pdgfrb*, *Epor*, *Erbb3*, *Il2rb* in aNSPCs treated with NAD+ (differentiation condition). Data are presented as the mean ± SEM. *n* = 3 independent experiments, unpaired *t*-test. (**G**–**I**) Western blot assay and the quantification results show that NAD+ exposure significantly decreased the level of p-Akt (ser473) while the level of total Akt was not affected. GAPDH was used as an internal control. Data are presented as the mean ± SEM. *n* = 3 independent experiments, unpaired *t*-test; *, *p* < 0.05; **, *p* < 0.01; ***, *p* < 0.001.

Given that RNA-seq data analysis showed that the altered expression of genes relating to ATP metabolism and the enrichment of DEGs in biological terms of NADP+ activity, we first performed an ELISA assay to measure the level of ATP; we observed that NAD+ exposure significantly increased the level of ATP (Figure 6A). In addition, NAD exposure led to a significant increase in the ratio of NADP+/NADPH (Figure 6B). These results suggest that NAD+ exposure alters the metabolic state of aNSPCs. 

RNA-seq data analysis also revealed that NAD+ exposure altered the FPKM values of multiple genes relating to the Akt signaling pathway (Figure 6C,D). Consistently, the RT-PCR assay results show that NAD+ exposure significantly reduced the expression of these Akt pathway-related genes including *Pdgfrb*, *Epor, Erbb3* and II2rb (Figure 6E,F). Furthermore, the Western blot assay and quantification results show that the level of phosphorylated Akt (p-Akt) was significantly reduced by NAD+ exposure but the level of total Akt was not altered (Figure 6G–I). Together, these results suggest that the Akt pathway is involved in the effects of NAD+ on the proliferation and differentiation of aNSPCs. 

## 4. Conclusions

In the present study, we showed that β-nicotinamide adenine dinucleotide hydrate (NAD+) exposure inhibited the proliferation and neuronal differentiation of aNSPCs, and induced the apoptosis of aNSPCs. NAD+ exposure also inhibited the morphological development of both newborn neurons generated by the differentiation of aNSPCs and primary hippocampal neurons. Mechanistically, we observed that NAD+ exposure altered the transcriptome and regulated the Akt signaling pathway. In summary, our study revealed a new role and a related mechanism of NAD+ in regulating neurogenesis and neural stem/progenitor cells development. 

Bioenergetics including the metabolism of diverse fuel molecules is a key factor for neuronal development, brain function and aging. Previous studies showed that the level of hippocampal NAD+ decreased with aging, and the inhibition of NAD+ biosynthesis could induce premature aging and recapitulate the defects of NSPCs induced by aging [18,28,29,30,31]. Previous studies also found that the supplementation of NAD+ could restore a youthful metabolism and could improve cognitive function and stem cell activity [28,32,33,34,35,36,37]. However, our present results show that the NAD+ supplement displayed inhibitory effects on the proliferation and neuronal differentiation of aNSPCs in vitro. These results suggest that the effects of NAD+ are influenced by diverse factors. Under pathological conditions, such as NAD+ depletion, the addition of NAD+ can attenuate neurotoxicity, improve aging-associated phenotype and play beneficial roles. However, under physiological conditions, the supplement of extra NAD+ could be detrimental. One limitation of our present study was that it was largely carried out in vitro, and an intensive in vivo study would be helpful to clarify this speculation. Collectively, these results suggest that NAD+ could have multi-faceted functions in the neuronal system, which might rely on specific conditions. 

As a fundamental molecule of the tricarboxylic citric acid (TCA) cycle, NAD+ has been shown to play important roles in multiple biological processes via diverse mechanisms [18,23]. NAD+ is essential for the homeostasis of mitochondria, which are impaired in neurodegenerative diseases including premature aging models and Alzheimer’s disease (AD) models [28,29,30,35]. NAD+ depletion also led to the dysregulation of neuronal calcium homeostasis and DNA repair capacity [28,29,30,38]. Of note, NAD+ could also modulate DNA demethylation, such as CEPBA, and could enhance its transcription in cancer cells [25]. Our present results show that NAD+ exposure did not affect the expression of *Cepba* but reduced the expression of multiple genes relating to Akt signaling pathway and the phosphorylation of Akt. Therefore, our study revealed a new layer of the mechanism in which NAD+ regulates neural stem cell function.

## Data Availability

The accession number for the RNA-seq data reported in this paper is GEO: GSE200494.

## Supplementary Materials

The following are available online at https://www.mdpi.com/article/10.3390/cells11081283/s1, Supplemental Figure S1: The examination of self-renewal and multipotent capabilities of aNSPCs. Supplemental Figure S2: NAD+ exposure affects the proliferation and differentiation of aNSPCs.
