# Supplementary figures and images for: NAD+ Modulates the Proliferation and Differentiation of Adult Neural Stem/Progenitor Cells via Akt Signaling Pathway
Source: Cells. 2022 Apr 9;11(8):1283. doi: 10.3390/cells11081283 (PMC9029130; doi:10.3390/cells11081283)

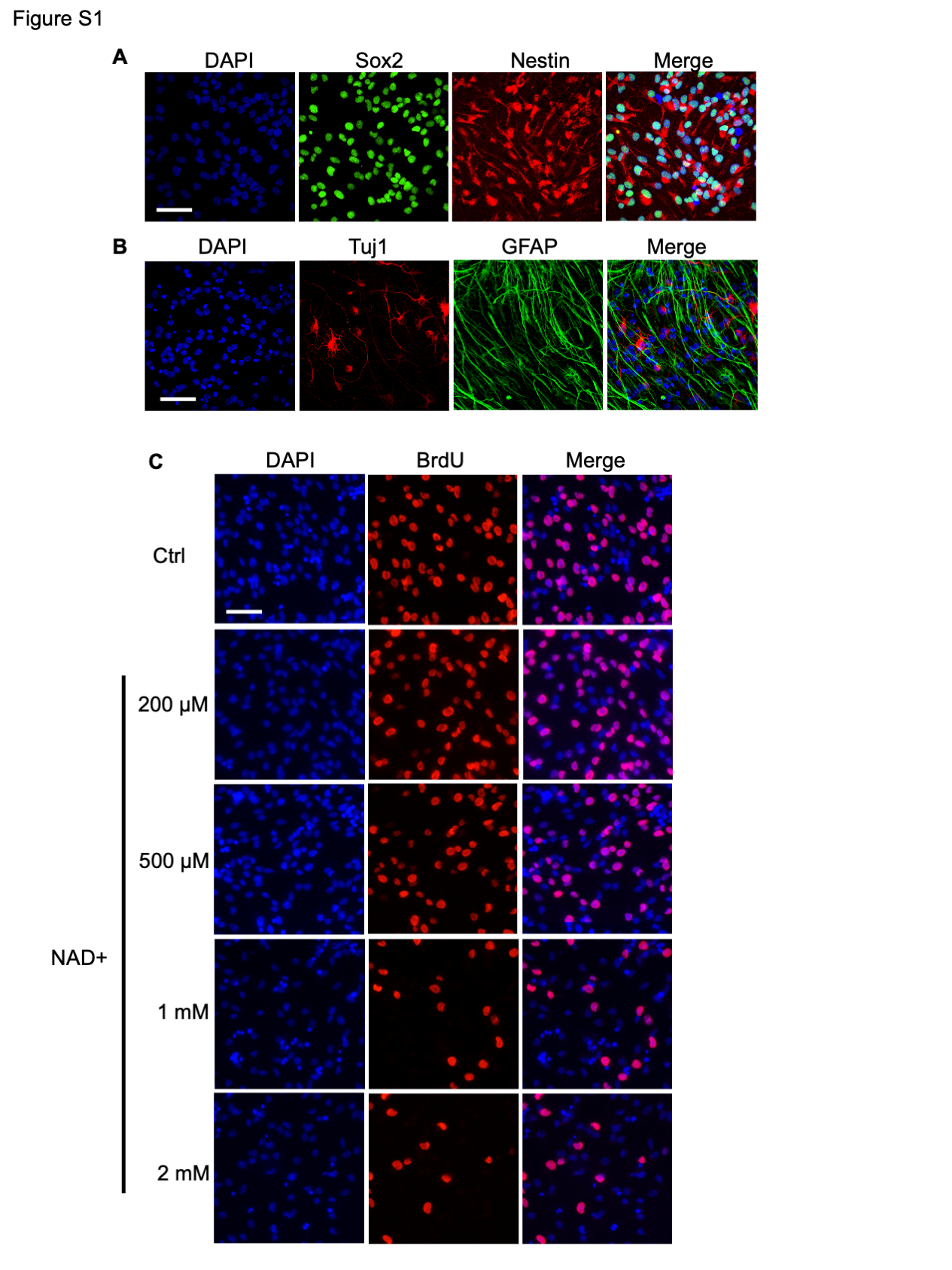

Supplement: Supplementary file 1 [file cells-11-01283-s001.zip › supplemental Figure S1.tiff]

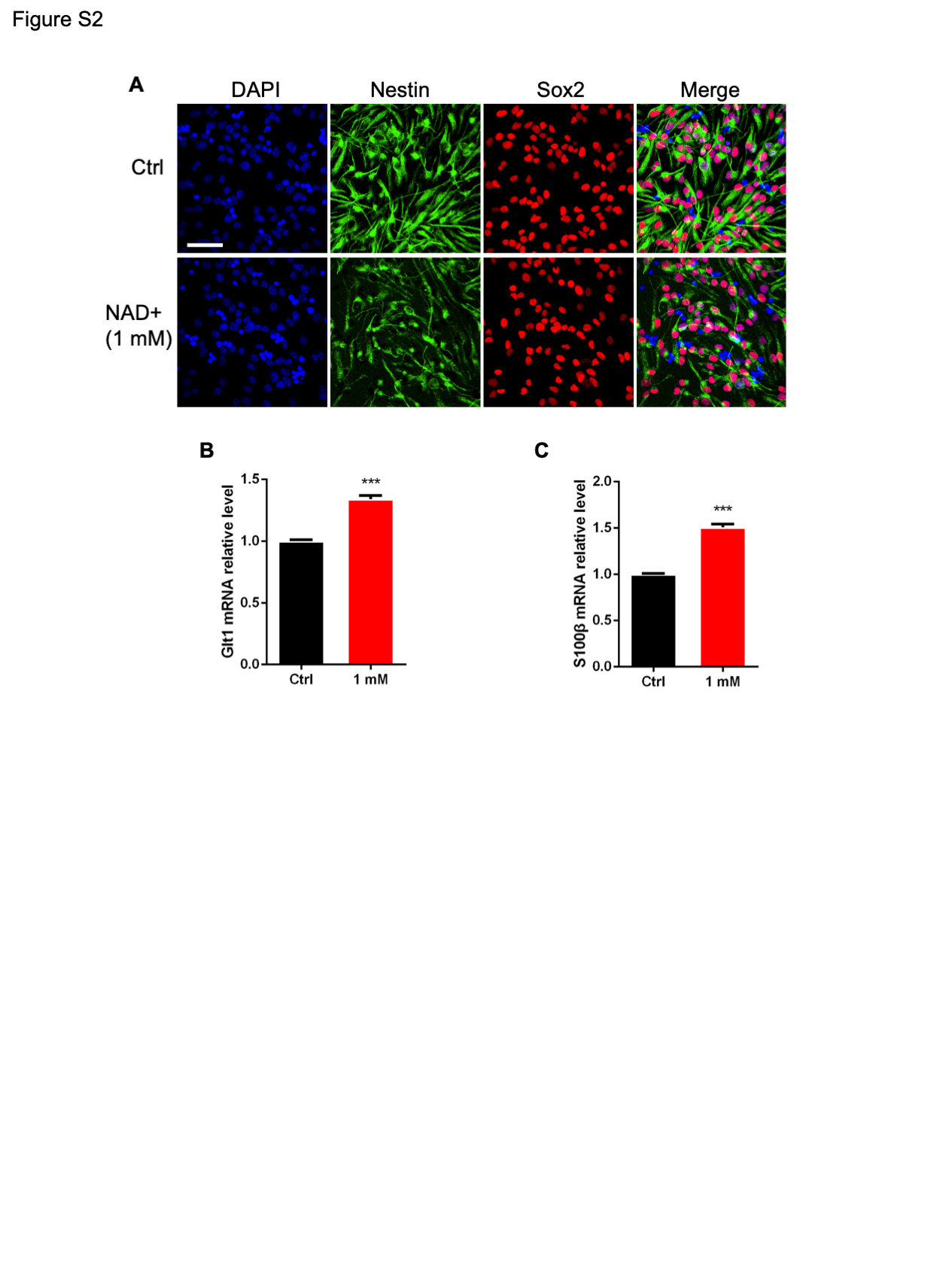

Supplement: Supplementary file 1 [file cells-11-01283-s001.zip › supplemental Figure S2.tiff]
